# Analysis of microbial compositions: a review of normalization and differential abundance analysis

**DOI:** 10.1038/s41522-020-00160-w

**Published:** 2020-12-02

**Authors:** Huang Lin, Shyamal Das Peddada

**Affiliations:** 1 Department of Biostatistics, University of Pittsburgh, 130 De Soto Street, Pittsburgh, PA 15261 USA; 2 Present Address: Biostatistics and Bioinformatics Branch, NICHD, NIH, Bethesda, MD USA

**Keywords:** Bacteria, Microbiology

## Abstract

Increasingly, researchers are discovering associations between microbiome and a wide range of human diseases such as obesity, inflammatory bowel diseases, HIV, and so on. The first step towards microbiome wide association studies is the characterization of the composition of human microbiome under different conditions. Determination of differentially abundant microbes between two or more environments, known as differential abundance (DA) analysis, is a challenging and an important problem that has received considerable interest during the past decade. It is well documented in the literature that the observed microbiome data (OTU/SV table) are relative abundances with an excess of zeros. Since relative abundances sum to a constant, these data are necessarily compositional. In this article we review some recent methods for DA analysis and describe their strengths and weaknesses.

## Introduction

Human oral and gut microbiome are estimated to have 45.6 million genes, which is ~2000-fold more genes than human genes^1^, therefore the microbiome is sometimes referred to as the “second genome”, or another “organ” of human body^2–4^. Hence it is not surprising that numerous diseases such as obesity^5^, inflammatory bowel diseases^6^ and HIV^7^ are associated or even caused by changes in the microbial ecosystem. For these reasons, understanding changes in the composition of microbiome under different conditions is important for studying human diseases.

For clarity, we begin by defining some important terms used in this paper and in the literature. The phrase absolute abundance of a taxon refers to the unobservable actual abundance of a taxon in a unit volume of an ecosystem, such as the gut. Accordingly, one could define absolute relative abundance of a taxon in a unit volume of an ecosystem as the ratio of the absolute abundance of the taxon to the total absolute abundance of all taxa in a unit volume of an ecosystem.

In practice, however, neither absolute abundance nor absolute relative abundance of a taxon in a unit volume of an ecosystem can be easily determined^8^. Although these parameters are typically not observable, the next-generation sequencing (NGS) technologies such as the 16S rRNA gene sequencing yield useful data for describing microbial compositions in an ecosystem. Following initial quality assessment/control steps, such as primer(s) removal, demultiplexing and quality filtering, the 16S amplicon sequences are either clustered into Operational Taxonomic Units (OTUs) representing the common working definition of bacterial species^9^ by OTU picking algorithms (e.g. UPARSE^10^), or grouped into Sequence Variants (SVs) using denoising algorithms (e.g. DADA2^11^ and Deblur^12^). After the construction of OTU or SV, these observed counts are typically organized into a large matrix referred to as the feature table. Some researchers or software packages such as QIIME2^13^ represent samples by columns and features (OTUs or SVs) by rows, but this representation is not universal. The observed counts of features (OTUs or SVs) represent observed abundances of taxa in the sample. Since abundances in a feature table represent only relative information regarding each taxa^8,14–18^, these are compositional data and thus reside inside a simplex^19^. Some researchers refer to these frequencies as relative abundances of taxa in a sample. However, in our terminology, relative abundance of a taxon in the sample is the fraction of the taxon observed in the feature table relative to the sum of all observed taxa corresponding to the sample in the feature table. Thus, by our terminology, the relative abundances sum to 1. In a recent paper by Lin and Peddada^20^, the authors refer to abundance of taxa in a feature table as “observed absolute abundance”, which is a confusing terminology and should be avoided. Instead they should have referred to it as “observed abundance”. Various terms used in this paper are summarized in Table 1. The notations described in statistical methods are summarized in Table 2.

**Table 1:** Definitions of key terminologies.

Term	Definition
Microbiota	Community of microscopic organisms.
Microbiome	Genes associated with the microbiota.
Amplicon	Product of PCR amplification.
High-throughput Sequencing	DNA sequencing approach that produces large amounts of sequence data rapidly at low cost.
OTU	Operational taxonomic unit: Group of DNA sequences with 97% similarity.
SV	Sequence variant: Individual DNA sequences recovered from a high-throughput marker gene analysis following the removal of spurious sequences generated during PCR amplification and sequencing.
Absolute abundance	Unobservable actual abundance of a taxon in a unit volume of an ecosystem.
Observed abundance	Observed counts of features (OTUs or SVs) in the feature table.
Relative abundance	The fraction of the taxon observed in the feature table relative to the sum of all taxa in the sample. It is between 0 and 1.
Feature Table	A matrix summarizing observed microbial abundances in the sample. Usually, columns represent samples and rows stand for OTUs or SVs.
Library Size	The total number of observed abundances for all taxa in a sample.
Microbial Load	The total number of (unobserved) absolute abundances for all taxa in a unit volume of an ecosystem.

**Table 2:** Summary of notations.

Notation	Description
*m*	Total number of taxa.
*n*	Total number of samples.
*p*	Total number of covariates.
*i*	Taxon index, *i* = 1, 2, …, *m*.
*j*	Sample index, *j* = 1, 2, …, *n*.
*k*	Index of covariates, *k* = 1, 2, …, *p*.
*x*_*j*_	Covariates of interest for the *j*th sample. ${x}_{j}={({x}_{j1},\ldots ,{x}_{\mathrm{jp}}\!)}^{T}$.
*A*_*ij*_^a^	Unobserved absolute abundance of *i*th taxon in a unit volume of ecosystem of *j*th sample.
*A*_⋅*j*_^a^	Microbial load in a unit volume of ecosystem of *j*th sample. ${A}_{\cdot j}=\mathop{\sum }\nolimits_{i = 1}^{m}{A}_{\mathrm{ij}\,}$.
*γ*_*ij*_^a^	Unobserved absolute relative abundance of *i*th taxon in a unit volume of ecosystem of *j*th sample.
*O*_*ij*_^a^	Observed abundance of *i*th taxon in a random specimen taken from a unit volume of ecosystem of *j*th sample.
*O*_⋅*j*_^a^	Library size of a random specimen taken from a unit volume of ecosystem of *j*th sample. ${O}_{\cdot j}=\mathop{\sum }\nolimits_{i = 1}^{m}{O}_{\mathrm{ij}\,}$.
*r*_*ij*_^a^	Observed relative abundance of *i*th taxon in a random specimen taken from a unit volume of ecosystem of *j*th sample.
*c*_*j*_^b^	For the *j*th sample, *c*_*j*_ represents the proportion of its ecosystem (unobserved absolute abundance) in a random specimen (observed abundance), thus ${c}_{j}=\frac{E({O}_{\mathrm{ij}\,}| {A}_{\mathrm{ij}\,})}{{A}_{\mathrm{ij}\,}}$. We shall refer to this constant as “sampling fraction”.
*y*_*ij*_^a^	$\mathrm{log}\,({O}_{\mathrm{ij}})$.
*d*_*j*_^b^	${\rm Represents \ the\ effect \ of \ the \ scaling \ parameter} \ {c}_{j} \ {\rm in \ log-scale}$

^a^Random variable.

^b^Parameter.

We define a taxon to be differentially abundant between two ecosystems if its mean absolute abundance is different between two ecosystems. It is important to distinguish between absolute and relative abundances of taxa in a unit volume of an ecosystem. The choice of parameter for statistical analysis is important and needs to be clearly stated. Often researchers are interested in identifying taxa that are different in mean absolute abundance per unit volume between two or more ecosystems^8^. The mean absolute abundance may not be the only criterion of interest. Researchers may consider other criteria such as differential ranking^8^. Furthermore, there are instances such as niche apportionment, where researchers are interested in identifying taxa that are different in mean absolute relative abundance per unit volume between two or more ecosystems. Thus, the choice of statistical parameter depends upon the scientific question of interest.

For each taxon *i* within sample *j*, the sampling fraction is the ratio of the expected abundance of taxon *i* within the *j*th sample to its absolute abundance in a unit volume of an ecosystem (e.g. gut) where the sample was derived from. The sampling fraction is assumed to be constant for all taxa within the *j*th sample. Thus the sampling fraction for the *j*th sample is given by the following expression involving the conditional expectation of the observed abundance *O*_*ij*_ given the unobservable absolute abundance *A*_*ij*_.

Definition 0.1 (Sampling fraction).

1 $${c}_{j}=\frac{E({O}_{\mathrm{ij}\,}| {A}_{\mathrm{ij}\,})}{{A}_{\mathrm{ij}\,}},$$

where

1. *O*_*ij*_ is the observed abundance of *i*th taxon in *j*th sample,
2. *A*_*ij*_ is the unobserved absolute abundance of *i*th taxon in the ecosystem of *j*th sample,
3. *c*_*j*_ is the sample-specific sampling fraction.

The problem underlying the differential abundance (DA) analysis of microbiome data is that while *O*_*ij*_ is known, *c*_*j*_ is unknown and can vary drastically from sample to sample. Consequently, the observed abundances are not comparable between samples. The goal of DA analysis described in this paper is to identify taxa whose mean absolute abundances, per unit volume, of an ecosystem are significantly different with changes in the covariate of interest (e.g. study groups).

**Fig. 1:**
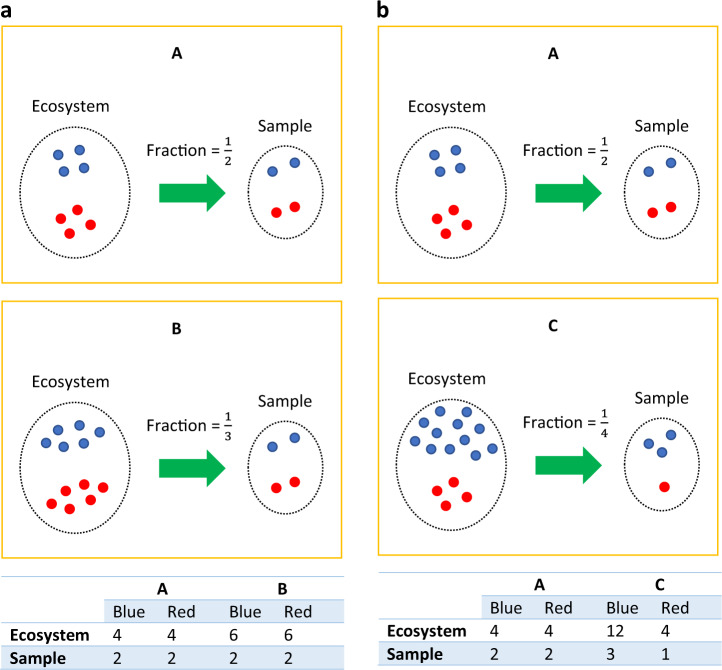
Microbiome data is represented by relative abundances, thus differential abundance analysis should account for the bias introduced by across-sample variations in sampling fractions. Sampling fraction is defined as the ratio of expected abundance in a sample to the corresponding absolute abundance in the ecosystem, which could be empirically estimated by the ratio of library size to the microbial load. **a** Differences in sampling fractions introduce false negatives. In this toy example, the microbial load for subject A in a unit volume of the ecosystem (e.g. a unit volume of gut) is 8 (4 blue + 4 red), while for subject B it is 12 (6 blue + 6 red). However, the samples taken from subject A and B have the same library size 4 (2 blue + 2 red), same observed abundance as well as the same relative abundance of blue and red taxa. Thus, one may mistakenly conclude that the blue and red taxa are not differentially abundant between two ecosystems, which is not the case in the two ecosystems. This false negative conclusion is caused by differences in the sampling fractions in the two samples. The sampling fraction in sample A is 1/2 and for B it is 1/3. **b** Differences in sampling fractions introduces false positives. Consider another subject C, who has the microbial load of 16 (12 blue + 4 red) in a unit volume of ecosystem. Given the same library size in sample C (3 blue + 1 red) as sample A, one may mistakenly conclude that both blue and red are differentially abundant between ecosystems A and C, while in fact, only the blue taxon is differentially abundant. Thus a normalization method must account for differences in sampling fractions to avoid such erroneous conclusions.

Similar to the toy example in ref. ^20^, Fig. 1 is a toy example consisting of ecosystems of three subjects A, B, and C with each having two taxa, the blue and red taxa varieties. A false negative may occur when comparing the ecosystems of A and B. Clearly, the true absolute abundance of each taxon is 50% more in subject B’s ecosystem as compared to subject A’s. However, they each have the same library size (4 each) in their respective samples (e.g. stool samples). Without considering the differential sampling fractions, one would falsely conclude that none of the taxa are differentially abundant in the two ecosystems. This erroneous conclusion would be avoided if one recognizes that we have a larger sampling fraction in the sample obtained from A’s ecosystem than from B’s ($\frac{1}{2}$ vs. $\frac{1}{3}$). Similarly, we get a false positive result when comparing ecosystems of A and C. In their ecosystems, blue is more abundant in C than in A (12 vs. 4), and both have the same amounts of red taxa (4 vs. 4). However, given that samples from A and C have same the library size, one may mistakenly conclude that both blue (2 vs. 3) and red taxa (2 vs. 1) are differentially abundant between A and C.

An important characteristic of a feature table is that it is typically sparse, sometimes as many as ~90% are zero entries^21^, which creates a challenge for analyzing rare taxa. A quick and simple strategy to deal with excess zeros is to add a small positive constant (e.g. 1) called pseudo-count^14,22^ to each cell of the feature table. The addition of a pseudo-count becomes necessary when using methods of analysis that require log transformation of the observed counts. Even though adding a pseudo-count is simple and widely used, the choice of the pseudo-count is ad hoc. Studies have shown that differential abundance or clustering results could be sensitive to the choice of pseudo count^23,24^. Although different values of pseudo counts have been discussed in the literature^23–26^, to the best of our knowledge, there is no consensus on how to choose the optimal value. Other strategies involve modeling zero counts by some probability models^21,27^. However, these methods may not be valid if the underlying assumptions do not hold. Instead of modeling zeros by parametric distributions, ANCOM-II^28^ attempts to provide a general framework to classify and identify zeros into three different types, which includes outlier zeros caused by some extraneous reasons such as the wrong data entry, structural zeros because of the nature of the study groups, i.e. some bacteria are not expected to belong to certain environments (e.g. a desert) but in others (e.g. a rain forest), and sampling zeros owing to insufficient library size. In our opinion, the zero counts problem is still an open problem and requires further investigation.

## Normalization methods

As we described intuitively in the introduction, an important obstacle for performing DA analysis is the unknown sampling fraction corresponding to each sample. It is critical to normalize the data to eliminate any bias due to differences in the sampling fractions. Thus, the primary objective of normalization is to transform the observed data so that expected differences in the mean absolute abundances between two ecosystems is not confounded by the differences in the sampling fractions. Failure to normalize the data will result in a systematic bias that increases the false discovery rate (FDR) and also possible loss of power in some cases.

### Rarefying

A traditional microbiome analysis workflow often involves rarefying^29–31^, or subsampling to a given depth, a practice in the field of ecology long before its use in microbiome surveys^32^. Samples are rarefied to deal with differences in library sizes. Note that the terms rarefying and rarefaction are used interchangeably in microbiome literature^33^. Rarefying was first recommended for microbiome data to deal with rare taxa^34^, which impact some measures of alpha and beta diversities^33^. Generally, the rarefying process includes the following steps:

1. Determine the minimum library size (${O}_{\min }$). Samples with library sizes smaller than ${O}_{\min }$ will be discarded,
2. Subsample taxa without replacement so that all samples have the same library size ${O}_{\min }$.

**Fig. 2:**
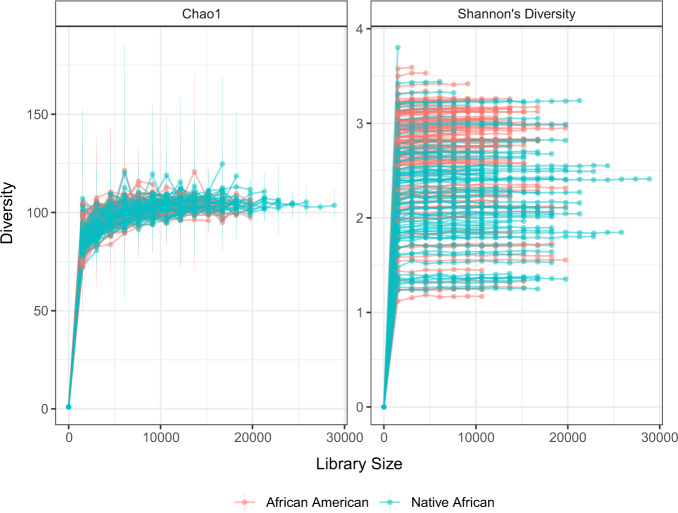
Rarefaction curves using the diet swap data^68^ at the genus level. The number of genera is 130, and the sample size is 222 (African American = 123, Native African = 99). *x* axis denotes the library size, and *y* axis represents the corresponding alpha diversity. Data are presented as mean values ± standard error (SE). It shows that regardless of the choice of diversity measures, as the increase of library size, the rarefaction curve starts to “level out” suggesting that the diversity of the samples has been fully observed.

One way to select the minimum library size is to create rarefaction curves^35^. Rarefaction curves represent diversity as a function of library size (Fig. 2). If lines of the plot appear to “level out” (i.e., approach a slope of zero) at certain library size along the x-axis, it indicates the diversity of the samples has been fully observed; otherwise, increasing the minimum library size would result in additional features. Originally, rarefaction curves were based on alpha diversities^35,36^. However, lately researchers have considered beta diversities^37,38^ as well. Although rarefying is well established and widely used in practice, in recent years there has been some discussion on the effects of rarefying on statistical tests for differential abundance analysis^33,39,40^. Some concerns discussed in the literature include:

1. The omission of available valid data,
2. The introduction of artificial uncertainty in the sub-sampling step,
3. The arbitrary selection of the minimum library size,
4. Challenges in estimating over-dispersion parameter.

### Scaling

Scaling is another popular method used for normalizing microbiome data. The basic idea is to divide the observed abundance in the feature table by a “scaling factor” or “normalization factor” to eliminate biases resulting from unequal sampling fractions. More precisely, scaling is defined as follows.

Definition 0.2 (Scaling microbiome data).

2 $${\tilde{O}}_{\mathrm{ij}\,}=\frac{{O}_{\mathrm{ij}\,}}{{s}_{j}},$$

where

1. ${\tilde{O}}_{\mathrm{ij}\,}$ is the normalized observed abundance for taxon *i* within sample *j*,
2. *s*_*j*_ is the scaling/normalization factor for sample *j*.

Comparing with the definition of sampling fraction (Eq. (1)), it is clear that an ideal scaling method should have scaling factor close to the unknown sampling fraction *c*_*j*_, i.e. *s*_*j*_ ≈ *c*_*j*_; or is approximately proportional to *c*_*j*_, i.e. *s*_*j*_ ≈ *c*_*j*_ × *c* for all *j*, where *c* is a constant.

Some commonly used normalization methods include Cumulative-Sum Scaling (CSS) implemented in metagenomeSeq^21^, Median (MED) in DESeq2^41^, Upper Quartile (UQ)^42^ and Trimmed Mean of M-values (TMM)^43^ in edgeR^44^ and Wrench^45^, and Total-Sum Scaling (TSS) which simply transforms the abundance table (feature table) into relative abundance table, i.e. scale by each sample’s library size. The authors of the user manual of edgeR^46^ state that to deal with the “RNA composition” effect, one should multiply the normalization factors with the corresponding library size to account for “effective library size”. Hence, Lin and Peddada^20^ also considered modified versions of UQ and TMM, denoted by “ELib-UQ” (Effective library size using UQ) and “ELib-TMM” (Effective library size using TMM) in their simulation studies. Since the literature is often not explicit regarding the mathematical formulas used by various methods, we provide some useful formulas in Table 3.

**Table 3:** Summary of different normalization methods.

Method	Sampling fraction estimate
ANCOM-BC	$\mathrm{log}\,({{\hat{c}}_{j}^{{\rm{ANCOM}}-{\rm{BC}}}})=\frac{1}{m}\mathop{\sum }\nolimits_{i = 1}^{m}({y}_{\mathrm{ij}\,}-{x}_{j}^{T}{{\hat{\beta }}_{i}})$
CSS	${\hat{c}}_{j}^{{\rm{CSS}}}=\frac{{s}_{j}^{\hat{l}}+1}{N}$
MED	${\hat{c}}_{j}^{{\rm{MED}}}={{\rm{median}}}_{i:{O}_{i}^{R}\ne 0}\frac{{O}_{\mathrm{ij}\,}}{{O}_{i}^{R}}$
UQ	${\hat{c}}_{j}^{{\rm{UQ}}}={{\rm{UQ}}}_{i:{O}_{\mathrm{ij}\,} \,{>}\,0}\left(\frac{{O}_{\mathrm{ij}\,}}{{O}_{\cdot j}}\right)$
TMM	${\mathrm{log}\,}_{2}({\hat{c}}_{j}^{{\rm{TMM}}})=\frac{{\sum }_{i\in G* }{w}_{\mathrm{ij}\,}{M}_{\mathrm{ij}\,}}{{\sum }_{i\in G* }{w}_{\mathrm{ij}\,}}$
Elib-UQ	${\hat{c}}_{j}^{{\rm{Elib}}-{\rm{UQ}}}={O}_{\cdot j}{\hat{c}}_{j}^{{\rm{UQ}}}$
Elib-TMM	${\hat{c}}_{j}^{{\rm{Elib}}-{\rm{TMM}}}={O}_{\cdot j}{\hat{c}}_{j}^{{\rm{TMM}}}$
Wrench	${\hat{c}}_{j}^{{\rm{Wrench}}}=\frac{1}{m}\mathop{\sum }\nolimits_{i = 1}^{m}{b}_{\mathrm{ij}\,}\frac{{r}_{\mathrm{ij}\,}}{{\bar{r}}_{i\cdot }}$
TSS	${\hat{c}}_{j}^{{\rm{TSS}}}={O}_{\cdot j}$

${\hat{\beta }}_{i}$ is obtained from ANCOM-BC algorithm.

*N* = an approximately chosen normalization constant.

$${s}_{j}^{\hat{l}}={\sum }_{i:{O}_{\mathrm{ij}\,}\,\le\, {q}_{j}^{\hat{l}}}{O}_{\mathrm{ij}\,}$$.

${q}_{j}^{\hat{l}}={\hat{l}}^{\mathrm{th}\,}$ quantile of sample *j*.

$${O}_{i}^{R}={\left(\mathop{\prod }\nolimits_{j = 1}^{n}{O}_{\mathrm{ij}\,}\right)}^{\frac{1}{n}}$$.

UQ(*X*) denotes the upper quartile of *X*.

${M}_{\mathrm{ij}\,}={\mathrm{log}\,}_{2}\left(\frac{{O}_{\mathrm{ij}\,}}{{O}_{\cdot j}}\right)-{\mathrm{log}\,}_{2}\left(\frac{{O}_{i{j}^{\prime}}}{{O}_{\cdot {j}^{\prime}}}\right)$, where ${j}^{\prime}$ is the reference sample.

${w}_{\mathrm{ij}\,}=\frac{{O}_{\cdot j}-{O}_{\mathrm{ij}\,}}{{O}_{\cdot j}{O}_{\mathrm{ij}\,}}+\frac{{O}_{\cdot {j}^{\prime}}-{O}_{i{j}^{\prime}}}{{O}_{\cdot {j}^{\prime}}{O}_{i{j}^{\prime}}}$, where ${j}^{\prime}$ is the reference sample.

G* represents a set of taxa that were not considered as extreme data for fold-change (M values) and average intensity (A values). Refer to Robinson and Oshlack^43^ for details.

*b*_*ij*_ represents the taxon-specific weight. Refer to Kumar et al.^45^ for details.

TSS is known to have a bias in differential abundance estimates^33,39,42,47^ since a few preferentially sampled measurements (e.g. taxa, genes) will have an undue influence on the relative abundance data. Change in the abundance of a single taxon can alter the relative abundances of all taxa. Generally, the FDR generated from TSS-based analyses is unacceptably large. The CSS^21^ in metagenomeSeq modifies TSS in a sample-specific manner to reduce biases resulting from preferentially sampled taxa. CSS assumes that observed abundances of samples should be roughly independent and identically distributed up to a specific quantile *l*. Thus, instead of normalizing each sample by its library size (which is also known as total sum), CSS selects the scaling factor to be the cumulative sum of observed abundances for each sample up to the *l*th quantile. This quantile is determined adaptively in a data-driven way, which relies on the change point of the distribution of cumulative sum switching from stability to instability. The Median normalization (MED) method used in DESeq2^41^ assumes that the taxon of median absolute abundance is not differentially abundant. Although it may be a valid assumption in gene expression studies where a large proportion of genes are not differentially expressed, it may not be a valid assumption in microbiome studies. Depending upon the application, a very large proportion of taxa may be differentially abundant between two or more study groups, especially when the data are analyzed at higher taxonomic classification levels (e.g. phylum, order, etc.). The Upper Quartile normalization (UQ) and the TMM used in edgeR have similar issues as MED in DESeq2. UQ assumes that the upper quartile of the observed abundances for each library is able to capture the invariant segment of the count distribution. However, choosing the most effective quantile is nontrivial^21,42,44,47–49^. Similar to MED, TMM is based on the hypothesis that most taxa are not differentially abundant. The scaling factor is calculated using a weighted trimmed mean of log abundance ratios by first trimming (by default) the taxa belong to upper and lower 30% *M* values (taxon-wise log-fold-change) or 5% *A* values (abundance level). Wrench^45^ assumes that the observed abundances are from a hurdle Log-Gaussian distribution. A robust location estimate of the Gaussian distribution leads to the desired scaling factor for each sample. However, Wrench currently implements strategies for categorical variable only, and the estimated scaling factor is essentially the average of ratios of relative abundances across taxa, which implicitly requires that a large proportion of taxa do not change across study groups, or the effect sizes of differentially abundant taxa are not too large.

One must exercise caution when using scaling methods. Most importantly, a scaling method is likely to overestimate or underestimate the fraction of zero counts depending on the corresponding library size of each sample^49,50^. This problem becomes more obvious for microbiome data since its feature table is typically sparse.

Recently a new method called Analysis of Compositions of Microbiome with Bias Correction (ANCOM-BC) was introduced by Lin and Peddada^20^ to address the problem of unequal sampling fractions. ANCOM-BC assumes that the observed abundance in a feature table is, in expectation, proportional to the unobservable absolute abundance of a taxon in a unit volume of the ecosystem. This proportion is defined as the sampling fraction and is allowed to vary from sample to sample. ANCOM-BC accounts for sampling fraction by introducing a sample-specific offset term in a linear regression model that is estimated from the observed abundance data. The offset term serves as the bias correction. Statistical properties of this approach have also been discussed in^20^.

**Fig. 3:**
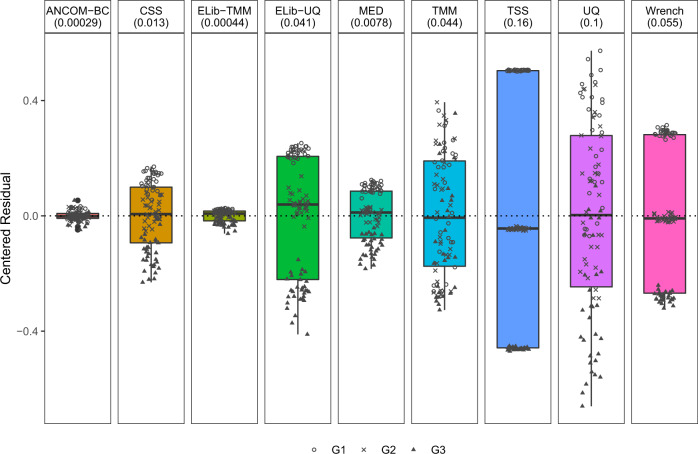
Box plot of residuals between true sampling fraction and its estimate for each sample. In the box plot, the lower and upper hinges correspond to the first and third quartiles (the 25th and 75th percentiles). The median is represented by a solid line within the box. The upper whisker extends from the hinge to the largest value (maxima) no further than 1.5 times Interquartile Range (IQR, distance between the first and third quartiles) from the hinge, the lower whisker extends from the hinge to the smallest value (minima) at most 1.5 times IQR of the hinge. Data beyond the end of the whiskers are called “outlying” points. *N* = 90 samples examined over three study groups (denoted by circle, cross, and triangle, with 30 samples per group) and the data points are overlaid in each box. Each facet title indicates the normalization method and its variance is provided within parenthesis. The microbial absolute abundances in the ecosystem are generated from the log-normal distribution. By comparing residuals across different groups, an ideal box-plot should display a narrow height (i.e. smaller variability) and samples from different groups should be inter-mixed and not display any systematic separation. We note that all existing methods have larger variances compared to ANCOM-BC, and TSS has the largest variance. Except ANCOM-BC, UQ, and TMM, we see from the plot that circles, cross, and triangles are systematically separated, which indicates that ELib-UQ, ELib-TMM, CSS, MED, and TSS do not account for systematic bias due to differences in sampling fractions across groups.

Extensive simulation studies using Poisson-Gamma model as well as some based on real data, were performed in^20^ to evaluate the performance of various normalization methods. Results reported in Fig. 3 of this article are similar to those provided in^20^, but in the present simulation study we have three groups, which are denoted by *G*_1_, *G*_2_, and *G*_3_ (see Supplementary Information for simulation settings). We compared all normalization methods using the centered residuals between true and estimated sampling fractions in log scale.

Definition 0.3 (Centered Residual).

3 $${h}_{j}={d}_{j}-{t}_{j}-\frac{1}{n}\mathop{\sum }\limits_{j\,=\,1}^{n}({d}_{j}-{t}_{j})$$

where

1. *dj* (see Table 2)
2. $${t}_{j}=\mathrm{log}\,{s}_{j}$$.

As noted at the beginning of this subsection, for each sample *j*, a reasonable scaling method should estimate scaling factors close to the true sampling fractions with possibly a constant shift across all samples. Not all scaling methods are expected to achieve this goal since many normalization methods were proposed solely to address the differences in library sizes (e.g. TSS). Failure to correct for differences in sampling fractions would lead to undesirable systematic bias in the test statistic, which can be identified by fitting a simple linear regression between centered residual *h*_*j*_ and the covariate of interest, such as *x*_*jk*_ (e.g. study groups):

4 $${h}_{j}={\alpha }_{0}+{\alpha }_{1}{x}_{\mathrm{jk}\,}+{e}_{j}.$$

The existence of systematic bias due to differences in sampling fractions may be determined by testing the null hypothesis *H*_0_: *α*_1_ = 0 against the alternative *H*_1_: *α*_1_ ≠ 0 or simply by drawing box plots of the centered residuals, as commonly done in linear regression diagnostics (Fig. 3). For an ideal normalization method, the box plot should display no pattern with respect to the covariate of interest, and the centered residuals should be randomly distributed around 0. As can be seen in the box plots provided in Fig. 3, except for ANCOM-BC, UQ, and TMM methods, for all other methods the groups *G*_1_, *G*_2_, and *G*_3_ cluster separately, indicating that in the estimation of sampling fractions, scaling factors estimated by these methods (with the exception of ANCOM-BC, UQ, and TMM) systematically differ by group labels. Furthermore, the box plot of ANCOM-BC had the shortest width, suggesting that it not only successfully estimates the true sampling fractions and eliminates bias due to its variability, but it also has the smallest variance which is not the case with other methods. This has a direct effect on the type I error and FDR as seen later in this paper and demonstrated in^20^.

### Log-ratio based methods

As an alternative to the above class of methods, several methods have been proposed in the literature that are inspired by Aitchison’s methodology for compositional data. These methods do not explicitly perform normalization such as the ones described above, since they convert the observed abundances to log-ratios within each sample. Thus, within each sample, by taking log-ratios of all taxa with respect to some common reference taxon or some suitable function of all taxa, these methods are intrinsically eliminating the effect of the sampling fraction. This class of methods include DR^8^, ANCOM^14^, and ALDEx2^51^. ALDEx2 uses a pre-specified taxon as a reference taxon and transforms the observed abundances to log ratios of the observed abundance each taxon relative to the reference taxon. Such a log-transformation of observed abundance data is called the additive log transformation (alr). Mathematically, it is defined as follows:

Definition 0.4 (additive log-ratio transformation (alr)^19^, ${\mathbb{S}^{m}\to \mathbb{R}^{m-1}}$).

5 $${\rm{alr}}({O_j})=\left[{\log}\,\left(\frac{{O}_{1j}}{{O}_{{i}^{\prime}j}}\right),\ldots ,{\log}\,\left(\frac{{O}_{{mj}\,}}{{O}_{{i}^{\prime}j}}\right)\right].$$

Thus, the alr transformation converts observed *m* dimensional observed abundance vector, representing the *m* taxa, that are in a simplex (i.e. sum to a constant), to a *m* − 1 dimensional data in the Euclidean space. A challenge with alr, and hence ALDEx2, is that the user needs to pre-specify the reference taxon. While this might be easy to do in some applications, it is generally a challenge when the number of taxa *m* is large such as when we are interested in performing DA analysis at the genus level. Although ANCOM is also based on alr transformation, it overcomes the above deficiency because it repeatedly applies the alr transformation by taking each of the *m* taxa to be a reference taxon one at a time. Thus, for each taxon, it performs *m* − 1 regressions. Hence, it overall fits *m*(*m* − 1) regression models.

To avoid the above challenges due to alr transformation, rather than using a pre-specified taxon as the reference taxon, one may consider the center of mass of all taxa as the reference. Thus, within each sample, for each taxon, the log-ratios are computed relative to the geometric mean of all taxa. This transformation is called the clr transformation. More precisely, it is defined as follows:

Definition 0.5 (centered log-ratio transformation (clr)^19^, ${\mathbb{S}^{m}\to \mathbb{U}^{m}}$).

6 $${\rm{clr}}({{\bf{O}}}_{{\bf{j}}})=\left[\mathrm{log}\,\left(\frac{{O}_{1j}}{g({{\bf{O}}}_{{\bf{j}}})}\right),\ldots ,\mathrm{log}\,\left(\frac{{O}_{\mathrm{mj}\,}}{g({{\bf{O}}}_{{\bf{j}}})}\right.\right],$$

where

1. *g*(*x*) is the geometric mean of *x*,
2. *U*^*m*^ = {(*u*_1_, …, *u*_*m*_) ϵ *R*^*m*^: *u*_1_ + … + *u*_*m*_ = 0} is a hyperplane in $\mathbb{R}^{m}$.

This transformation to a real space again makes the implementation of unconstrained statistical methods possible. clr transformation is an isometry, but sum of the transformed values equals to 0, leading to a degenerate distribution.

The alr transformation is not isometric and clr is not an isomorphism. The isometric log-ratio transformation (ilr)^25^ (also known as balance) is both an isomorphism and an isometry, and consequently orthonormal coordinates can be defined using this transformation.

Definition 0.6 (isometric log-ratio transformation (ilr), $\mathbb{S}^{m}\to \mathbb{R}^{m-1}$).

7 $${\rm{ilr}}({{\bf{O}}}_{{\bf{j}}})={\rm{clr}}({{\bf{O}}}_{{\bf{j}}}){\Psi }^{T},$$

where Ψ is a (*m* − 1, *m*) orthonormal basis.

There are multiple ways to construct orthonormal bases. Typically, if a bifurcating tree is given then we can construct a basis from the internal nodes in the tree. Each element in the ilr transformed data is of the following form:

8 $${b}_{l}=\sqrt{\frac{| {l}_{L}| | {l}_{R}| }{| {l}_{L}| +| {l}_{R}| }}\mathrm{log}\,\left[\frac{g({l}_{L})}{g({l}_{R})}\right],$$

where

1. *b*_*l*_ is the balance at internal node *l*,
2. *l*_*L*_ is the set of relative abundances contained in the left subtree at internal node *l*,
3. *l*_*R*_ is the set of relative abundances contained in the right subtree at internal node *l*,
4. ∣*l*_*L*_∣ is the number of taxa contained in *l*_*L*_,
5. ∣*l*_*R*_∣ is the the number of taxa contained in *l*_*R*_,
6. *g*(*x*) is the geometric mean of *x*.

## Methods of differential abundance analysis

A number of procedures have been introduced and used in the literature for identifying differentially abundant taxa. One common approach is to apply a nonparametric test (e.g. the Mann–Whitney/Wilcoxon rank-sum test for two sample classes; the Kruskal–Wallis test for multiple sample classes) after normalizing the feature table. Unfortunately, these standard nonparametric tests do not take into account the compositional structure of microbiome data.

### RNA-seq based methods: edgeR and DESeq2

As alternatives to standard nonparametric tests, many parametric models have been proposed in the literature based on transcriptomics data, such as the RNA-Seq data, for testing differences across study groups. Among them, DESeq2^41^ and edgeR^44^ are two popular methods. These methods model the observed abundances using negative binomial (NB) distribution after normalizing data with corresponding scaling methods to account for differences in sampling fractions. Thus *O*_*ij*_ are modeled using the a negative binomial distribution as follows:

9 $${O}_{\mathrm{ij}\,} \sim {\rm{NB}}({s}_{j}{\mu }_{i},{\phi }_{i}),$$

where

1. *s*_*j*_ is the scaling factor for sample *j*,
2. *μ*_*i*_ is the mean absolute abundance (in ecosystem) for taxon *i*,
3. *ϕ*_*i*_ is the dispersion parameter for taxon *i*.

Introduction of the dispersion parameter *ϕ*_*i*_ is inspired by mean-variance dependence in count data (e.g. RNA-Seq, microbiome data), and recognizing that the variance is typically larger than mean especially when the mean value is large. Thus, the variance of the observed abundance is modeled as follows:

10 $${\rm{Var}}({O}_{\mathrm{ij}\,})={s}_{j}{\mu }_{i}+{\phi }_{i}{s}_{j}^{2}{\mu }_{i}^{2}.$$

The NB distribution is more appropriate for modeling these types of count data than the Poisson distribution because it provides greater flexibility in modeling the variance. We remind the readers that by conditioning independent Poisson random variables on the total count results in multinomial distribution^52,53^.

The estimation of the dispersion parameter is critical for both edgeR as well as DESeq2. Based on the assumption that taxa with similar observed abundances also share similar variances, edgeR estimates the taxon-wise dispersion by conditional maximum likelihood^54^, and then shrinks the dispersion estimate for each taxon towards a common estimate of taxa with similar observed abundances using an empirical Bayes procedure^55^. Similarly, DESeq2 first estimates the taxon-wise dispersion by maximum likelihood estimation, and then fits the dispersion trend combining all individual estimates, and finally shrinks the taxon-wise dispersion estimates towards the values predicted by the trend curve using an empirical Bayes approach.

**Fig. 4:**
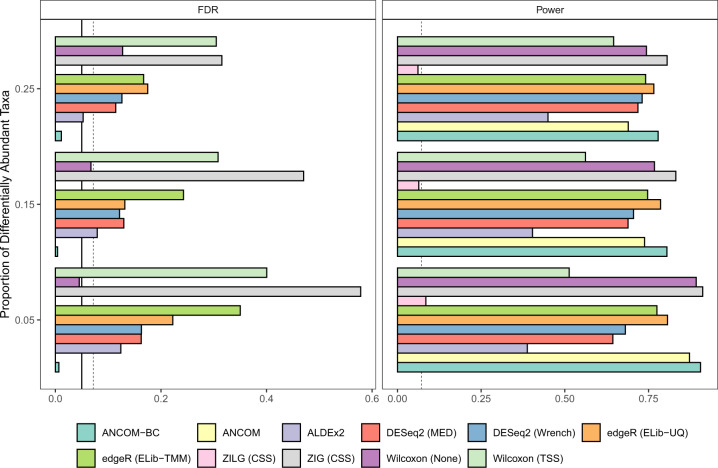
False Discovery Rate (FDR) and power comparisons using synthetic data. The FDR and power of various differential abundance (DA) analyses (two-sided) are shown in **a**, **b**, respectively. The number of taxa is set to be 200, and the sample size equals to 60, with 30 samples per group. The microbial absolute abundances in the ecosystem are generated from the log-normal distribution. The *y*-axis denotes patterns of proportion of differential abundant taxa, ranging from 0.05 to 0.25. The solid vertical line is the 5% nominal level of FDR, and the dashed vertical line denotes 5% nominal level plus one standard error (SE). Legend on the bottom indicates the color for each method and the normalization method is provided within parenthesis. By default, ANCOM-BC implements Holm-Bonferroni^70^ method and other DA methods implement BH procedure^57^ to adjust for multiple comparisons. By detecting differentially abundant taxa between two groups, results show that only ANCOM and ANCOM-BC control the FDR under the nominal level (5%) while maintaining power comparable to other methods. Gaussian model version of metagenomeSeq has highly inflated FDR, while the log Gaussian version has substantial loss of power, sometimes well below 5%.

While both methods are generally very reasonable and appropriate for gene expression data, they seem to perform poorly for microbiome data. This is largely because, as stated earlier, the normalization methods used by these two methods intrinsically assume that a very small fraction of taxa are differentially abundant. This assumption is not necessarily valid for microbiome data. As a consequence, the test statistics used by these methods are intrinsically biased under the null hypothesis. As demonstrated analytically as well as empirically in Lin and Peddada^20^, and reproduced here empirically using similar log-normal distribution based simulation settings (Fig. 4, see Supplementary Information for simulation settings), the bias in the test statistic results in inflated FDRs for these methods. What is worse, because of the bias, as the sample size increases, the FDR increases for these methods^20^. Similar phenomena were reported by Weiss et al.^39^. When dealing with population studies, it is important to recognize that there is variability within subject and there is variability between subjects in the population. In simple terms, observed abundance of a taxon from a subject may vary from stool sample to stool sample obtained from the same subject. This is within subject variation. Hence when calculating variability in measurements of random subject, one needs to take into account variation within as well as between subjects. This results in over-dispersion^33^. While it is important to account for this over-dispersion, it does not correct the intrinsic bias due to differential sampling fractions noted above. RNA-seq inspired methods do not perform well for microbiome data even after correcting for the over-dispersion parameter.

### MetagenomeSeq

Instead of using a negative binomial model, an alternative mixture model based on zero-inflated Gaussian (ZIG) is implemented in metagenomeSeq^21^, where excess zeros due to both sampling zeros and structural zeros are accounted by a probability mass, and the Gaussian distribution modeling the non-zero observed abundances. The framework can be summarized as follows:

11 $$\begin{array}{l}{y}_{\mathrm{ij}\,}={\mathrm{log}\,}_{2}({O}_{\mathrm{ij}\,}+1),\\ {f}_{\mathrm{zig}\,}({y}_{\mathrm{ij}\,},{O}_{\cdot j},{\mu }_{i},{\sigma }_{i}^{2})={\pi }_{j}({O}_{\cdot j}){I}_{\{0\}}({y}_{\mathrm{ij}\,})+[1-{\pi }_{j}({O}_{\cdot j})]\phi ({y}_{\mathrm{ij}\,},{\mu }_{i},{\sigma }_{i}^{2}),\\ {\mu }_{i}={\eta }_{i}{\mathrm{log}\,}_{2}(\frac{{s}_{j}^{\hat{l}}+1}{N})+{{\beta }_{{\bf{i}}}}^{T}{{\bf{x}}}_{{\bf{j}}},\end{array}$$

where

1. *N* is a normalization constant,
2. $\hat{l}$ is determined by CSS normalization,
3. ${q}_{j}^{\hat{l}}$ is the ${\hat{l}}^{\mathrm{th}\,}$ quantile of observed abundances for sample *j*,
4. $${{s}_{j}^{\hat{l}}=\mathop{\sum }\nolimits_{i:{O}_{\mathrm{ij}}}\le {{q}_{j}^{\hat{l}}}^{\hat{l}}{O}_{\mathrm{ij}}}$$.

However, as shown in our benchmark simulations (Fig. 4) as well as in other previously published simulation studies^14,33,39^, although metagenomeSeq has marginally higher powers than most of the other DA methods, it is subject to unreasonably high FDRs even though the observed abundances are normalized by their built-in scaling method (CSS). Furthermore, the problem of FDR inflation gets worse when sample size or the effect size (i.e. fold change of mean absolute abundances) increases^20,39^. It is also worth pointing out that metagenomeSeq was the only method, among all parametric models, that increases FDR when applied to rarefied data^33,39^. This is likely due to its zero-inflated model which requires the input of precise library sizes to capture the zero proportions.

Note that the authors of metagenomeSeq modified their procedure and recommended replacing zero-inflated Gaussian (ZIG) mixture model by zero-inflated Log-Gaussian (ZILG) mixture model for DA analysis. Although switching to zero-inflated Log-Gaussian distribution improves the FDR control, the procedure becomes extremely conservative, with FDR close to zero and a substantial loss of power in our simulations (Fig. 4) and in ref. ^20^.

### ALDEx2

It is based on the original version of ANOVA-Like Differential Expression (ALDEx) analysis^56^. It was proposed as a compositional data analysis tool that is applicable to three different types of data: RNA-Seq, ChIP-Seq, and 16S rRNA gene sequencing^51^. By acknowledging these high-throughput sequencing data are fundamentally compositional, the methodology of ALDEx2 can be summarized as follows:

1. The observed abundances are converted to relative abundances by Monte Carlo (MC) sampling from the Dirichlet distribution with the addition of a uniform prior. The MC sampling is repeated for *K* times (*K* = 128 times by default), thus essentially, for each taxon *i* in sample *j*, the observed abundance *O*_*ij*_ is represented by a vector of MC samples of relative abundances ${({r}_{\mathrm{ij}\,}^{(1)},\ldots ,{r}_{\mathrm{ij}\,}^{(K)})}^{T}$,
2. Within each sample *j* and each MC Dirichlet realization *k*, *k* = 1, …, *K*, the relative abundance vector ${({r}_{1j}^{(k)},\ldots ,{r}_{\mathrm{mj}\,}^{(k)})}^{T}$ is clr transformed,
3. Significance test (Welch’s *t*-test or Wilcoxon test) is performed on each taxon in the vector of clr transformed values. Since there are a total of *K* MC Dirichlet samples, each taxon will result in *K* *p*-values.
4. Each resulting *p*-value is corrected using the B–H^57^ procedure, and the expected adjusted p-value for each taxon is reported by taking the empirical mean of *K* adjusted *p*-values.

The ALDEx2 was designed to identify differential abundances of features (genes, taxa, or genomic segments), relative to the geometric mean abundance, between two or more groups. As reported in the simulation study described in this paper (Fig. 4) ALDEx2 not only generally exceeds the nominal level of FDR (5%), but also has substantially smaller power as compared to competing DA methods. Similar results were also reported in Morton et al.^8^.

### ANCOM

Analysis of composition of microbiomes (ANCOM)^14^ is an alr based methodology, which accounts for the compositional structure of microbiome data. Given a total of *m* taxa, ANCOM relies on two assumptions as follows.

*Assumption 0.1*: The mean log absolute abundance (in the ecosystem) of 2 taxa are not different.

*Assumption 0.2*: The mean log absolute abundance (in the ecosystem) of all *m* taxa do not differ by the same amount between two study groups. For example, suppose the absolute abundance of *m* taxa for a subject in group 1 (C-section born babies) are *A*_1_, *A*_2_, …, *A*_*m*_ and suppose the absolute abundance of taxa for a subject in group 2 (vaginally born babies) are *B*_1_, *B*_2_, …, *B*_*m*_. Then *B*_*i*_ ≠ *CA*_*i*_, for all *i* = 1, 2, …, *m*. Thus, not all taxa are changing by the same constant *C*.

Note that the first assumption made by ANCOM is substantially weaker than the assumptions made by DESeq2 and edgeR, which require very “few” taxa to be differentially abundant.

Under the above assumptions, together with the fact that ANCOM performs all possible DA analyses by successively using each taxon as a reference taxon, the authors proved that one can test the null hypothesis regarding mean log absolute abundance in a unit volume of an ecosystem using relative abundances.

For the *i*th taxon and *j*th sample, ANCOM uses standard ANOVA model formulation:

12 $$\mathrm{log}\,\frac{{r}_{\mathrm{ij}\,}^{(g)}}{{r}_{{i}^{\prime}j}^{(g)}}={\alpha }_{i{i}^{\prime}}+{\beta }_{i{i}^{\prime}}^{(g)}+\mathop {\sum}\limits_{k}{x}_{\mathrm{jk}\,}{\beta }_{i{i}^{\prime}k}+{\epsilon }_{i{i}^{\prime}j}^{(g)},$$

where

1. ${i}^{\prime}$ is the reference taxon, ${i}^{\prime}\,\ne \,i=1,2,\ldots ,m$,
2. *g* = 1, 2, …, *G* is the number of study groups.

By virtue of Assumption 0.1 and Assumption 0.2, to test whether a taxon *i* is differentially abundant according to a factor of interest with *G* levels, it is equivalent to test:

$$\begin{array}{l}{H}_{0(i{i}{\prime})}:{\beta }_{i{i}{\prime}}^{(1)}=\ldots ={\beta }_{i{i}{\prime}}^{(G)}=0,\\ {H}_{1(i{i}{\prime})}:\,\text{Not all}\,{\beta }_{i{i}{\prime}}^{(g)}\,\text{equals to}\,\,0,\end{array}$$

for every $$i\,\ne\,{i}^{\prime}$$.

*P*-values from $\frac{m(m-1)}{2}$ distinct null hypotheses ${H}_{0(i{i}^{\prime})}$, $i\,\ne\,{i}^{\prime}$ are adjusted using a multiple testing correction procedure such as the Benjamini-Hochberg (BH) procedure^57^ or Bonferroni correction^58,59^. For each taxon, the number of rejections, denoted by *W*_*i*_, is counted, and ANCOM makes use of the empirical distribution of {*W*_1_, *W*_2_, …, *W*_*m*_} to determine the cut-off value of significant taxon. The rule of thumb is, when the value of *W*_*i*_ is large, then it is more likely that taxon *i* is differentially abundant. The authors recommend using 70th percentile of the *W* distribution as the empirical cut-off value. However, the ANCOM outputs results from different cutoffs such as the 60th to 90th percentile and lets the user select the threshold of their interest.

As shown in the simulation studies (Fig. 4) as well as in^14,20^, using the 70th percentile of *W* distribution as the cut-off, ANCOM successfully controls the FDR under the nominal level (5%) while maintaining adequate power. However, ANCOM can be computationally intensive since for each taxon, it performs alr transformation using all remaining taxa. The computation time scales up quadratically with the number of taxa. Additionally, the statistical decision made by ANCOM depends on the quantile of its test statistic *W*, rather than p-values, which some researchers find it difficult to interpret.

### DR

Differential Ranking (DR)^8^ exploits the fact that the ranks of relative differentials (i.e. log ratio between absolute relative abundances) are identical to the ranks of absolute differentials (i.e. log ratio between absolute abundances). They estimate relative differentials using a linear regression where relative abundances are alr transformed. The regression coefficients corresponding to different taxa are ranked in order to determine the most important to the least important taxa.

The DR model can be summarized as follows:

13 $$\begin{array}{l}{\beta }_{\mathrm{ik}\,} \sim N(0,{\mu }_{\beta }),\\ {{\bf{r}}}_{{\bf{j}}}={{\rm{alr}}}^{-1}({\beta }_{{\bf{i}}}^{T}{{\bf{x}}}_{{\bf{j}}}),\\ {{\bf{A}}}_{{\bf{j}}} \sim {\rm{Multinomial}}({{\bf{r}}}_{{\bf{j}}}),\end{array}$$

where

1. **x**_**j**_ is the vector of covariates of interest (e.g. study groups) for the *j*th sample,
2. **r**_**j**_ is the vector of observed relative abundances for the *j*th sample,
3. **A**_**j**_ is the vector of absolute abundances in the ecosystem for the *j*th sample.

The model parameters are estimated using a maximum a posteriori priori (MAP) estimation by stochastic gradient descent.

To understand the implementation of the DR procedure, consider a simple example where the true absolute relative abundance is known. Suppose there are only two samples belonging to two groups (e.g. control vs treatment) and the unobserved absolute abundance is linearly related with the group effect in log scale, i.e.:

14 $$\mathrm{log}\,{A}_{\mathrm{ij}\,}={\alpha }_{i0}+{\alpha }_{i1}I \,(j\in {\rm{group}}\,1).$$

Suppose sample *j*_1_ is in group 1 and sample *j*_2_ is in group 2, then from (Eq. 14) we have

15 $$\mathrm{log}\,{A}_{i{j}_{1}}-\mathrm{log}\,{A}_{i{j}_{2}}={\alpha }_{i1}.$$

Denoting the true absolute relative abundances by *γ*_*ij*_ and ${\gamma }_{{i}^{\prime}j}$ one can write down the DR model (Eq. 13) as:

16 $$\mathrm{log}\,\frac{{\gamma }_{\mathrm{ij}\,}}{{\gamma }_{{i}^{\prime}j}}=\mathrm{log}\,\frac{{A}_{\mathrm{ij}\,}}{{A}_{{i}^{\prime}j}}={\beta }_{i0}+{\beta }_{i1}.$$

where ${i}^{\prime}$ is the reference taxon. Thus,

17 $$\begin{array}{*{20}{l}}{\log}\,\frac{{\gamma }_{i{j}_{1}}}{{\gamma }_{{i}^{\prime}{j}_{1}}}-{\log}\,\frac{{\gamma }_{i{j}_{2}}}{{\gamma }_{{i}^{\prime}{j}_{2}}}={\log}\,\frac{{A}_{i{j}_{1}}}{{A}_{{i}^{\prime}{j}_{1}}}-{\log}\,\frac{{A}_{i{j}_{2}}}{{A}_{{i}^{\prime}{j}_{2}}}\\ ={\log}\,{A}_{i{j}_{1}}-{\log}\,{A}_{i{j}_{2}}-({\log}\,{A}_{{i}^{\prime}{j}_{1}}-{\log}\,{A}_{{i}^{\prime}{j}_{2}})\\ ={\beta }_{i1}.\end{array}$$

Comparing (Eq. 15) with (Eq. 17), it is clear that although *β*_*i*1_ ≠ *α*_*i*1_, due to the bias term $\mathrm{log}\,{A}_{{i}^{\prime}{j}_{1}}-\mathrm{log}\,{A}_{{i}^{\prime}{j}_{2}}$. However, since the bias term is constant for taxon *i*, the rank of *β*_*i*1_ is same as the rank of *α*_*i*1_.

Thus, unlike typical DA methods in which the estimated coefficient reflects the change in absolute abundances, the interpretation of DR results requires care because it is based on the ranks. Due to the presence of the microbial load bias ($\mathrm{log}\,{A}_{{i}^{\prime}{j}_{1}}-\mathrm{log}\,{A}_{{i}^{\prime}{j}_{2}}$ in the above example), a positive valued coefficient from DR model does not necessarily mean that the absolute abundance has increased. Similarly, a zero valued coefficient does not imply the absolute abundance of the corresponding taxon has not changed. Nevertheless, based on the ranks of coefficients, one can focus on taxa with high or low ranks since they are the ones that are potentially increasing or decreasing the most in absolute abundances relative to other taxa.

Note that since different reference taxon in the alr transformation of DR model will lead to the same result regarding the ranks, DR is robust to the choice of reference taxon.

### ANCOM-BC

Analysis of compositions of microbiomes with bias correction (ANCOM-BC)^20^ models the observed abundances using an offset-based log-linear model.

18 $${y}_{\mathrm{ij}\,}={d}_{j}+{{\beta }_{{\bf{i}}}}^{T}{{\bf{x}}}_{{\bf{j}}}+{\epsilon }_{\mathrm{ij}\,},$$

where

1. ${y}_{\mathrm{ij}\,}=\mathrm{log}\,{O}_{\mathrm{ij}\,}$ is the log observed abundance,
2. *dj* (see Table 2)

In this set-up, the zero counts are handled using the methodology described in Kaul et al.^28^. This formulation explicitly tests the hypothesis regarding differential absolute abundance of individual taxon while estimating sample-specific sampling fractions and correcting the bias appropriately. As demonstrated in our simulation studies, ANCOM-BC not only controls the FDR very well, but also competes very well with other methods in terms of power (Fig. 4). Furthermore, unlike any of the existing methods, ANCOM-BC provides valid confidence intervals for differential abundance of individual taxa between two study groups and also provides a valid *p*-value^20^. Since it has a linear regression framework, it allows for repeated measurement designs as well as covariate adjustments. ANCOM-BC can also be extended to describe patterns of differential abundance in multiple study groups such as time course or dose-response studies^20^.

**Fig. 5:**
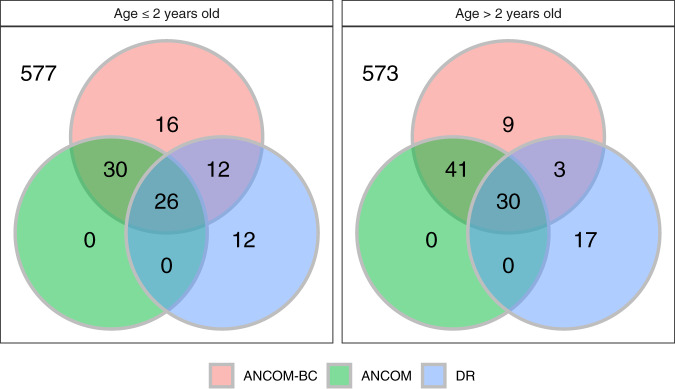
Venn diagrams representing consistency of differentially abundant genera identified by ANCOM-BC, ANCOM, and DR. The global gut microbiota data^60^ is used to make the Venn diagram. The dataset contains 673 genera, with subjects from Malawi (MA, *n*_1_ = 114), USA (US, *n*_2_ = 317), and Venezuela (VEN, *n*_3_ = 99). We compare the absolute abundance of different genera for (1) Subjects who are less than or equal to 2 years old with sample size (MA:US:VEN) = (47:50:27) and (2) Subjects who are greater than 2 years old with sample size (MA:US:VEN) = (36:260:70). ANCOM-BC and ANCOM generally have large overlap of significant genera.

As a benchmark analysis, we also compared significant genera identified by ANCOM-BC, ANCOM, and DR using the global gut microbiota data^60^. This data set consists of 11,905 OTUs obtained from fecal samples of subjects in the USA (*n* = 317), Malawi (*n* = 114), and Venezuela (*n* = 99). We first subdivided the data into two age strata “≤2 years” and “>2 years”. This stratification was performed because it is expected that microbial composition of infants changes when they switch over from breast milk (or formula milk) to solid food^7^. The sample sizes in the two age categories (≤2 years, >2 years) for Malawi (MA), USA (US) and Venezuela (VEN) are (47, 36), (50, 260), and (27, 70), respectively. Note that samples with missing values of age were discarded in the downstream analysis. Without a hard threshold available for DR, as suggested in the original paper^8^, we investigated the highest/lowest ranks of genera by selecting the top 25 and bottom 25 genera in terms of rank order of regression parameter estimates. As seen in Fig. 5, the three methods generally have a large number of overlapping genera, with ANCOM-BC and ANCOM having more taxa in common that are differentially abundant. While implementing ANCOM, we used the 70th percentile of the distribution of *W* as the cut-off. Note that the DR method was applied with all hyper-parameters of the multinomial model set to their default values in the algorithm which can be further tuned.

### Balance-based methods

A variety of methods have been proposed in the literature that are based on balances described earlier in this paper. Some examples include gneiss^18^, phylofactorization^61,62^, PhILR^63^, and selbal^64^. Although the balance-based methods were not explicitly designed for performing formal statistical DA analyses for individual taxon, they are often used for that purpose.

To overcome the challenges posed by the compositional structure of 16S rRNA data for identifying individual differentially abundant taxa, gneiss^18^ was developed to identify taxa distribution across different covariates with the help of balances. The balances (Eq. (8))^65,66^ are useful to infer meaningful properties of sub-communities. Gneiss aims to associate the effect of parameter of interest to the matrix of balances:

Definition 0.7 (gneiss model).

19 $${b}_{\mathrm{jl}\,}={\beta }_{{\bf{l}}}^{T}{{\bf{x}}}_{{\bf{j}}},$$

where

1. *b*_*jl*_ represents the balance for sample *j* at node *l*,
2. ${\beta }_{{\bf{l}}}={({\beta }_{l1},\ldots ,{\beta }_{\mathrm{lp}\,})}^{T}$ represents a vector of coefficients,
3. ${{\bf{x}}}_{{\bf{j}}}={({x}_{j1},\ldots ,{x}_{\mathrm{jp}\,})}^{T}$ represents the measures for covariates.

Gneiss methodology is very flexible and can be broadly used for determining niches of microbes in various sub-communities. Thus, it is a very useful method for discovering niche differentiation in microbes.

Similar to gneiss, phylofactorization^61,62^ is not designed for the DA analysis as defined in this paper, but it focuses on the comparison between clades with a clear phylogenetic interpretation. It is based on a greedy algorithm which sequentially selects edges, instead of nodes or splits in a phylogeny, whose ilr basis element maximizes a pre-specified objective function (e.g. the percentage of variation explained). Therefore, besides comparing sister clades, phylofactorization compares the relative abundances between all other clades.

**Fig. 6:**
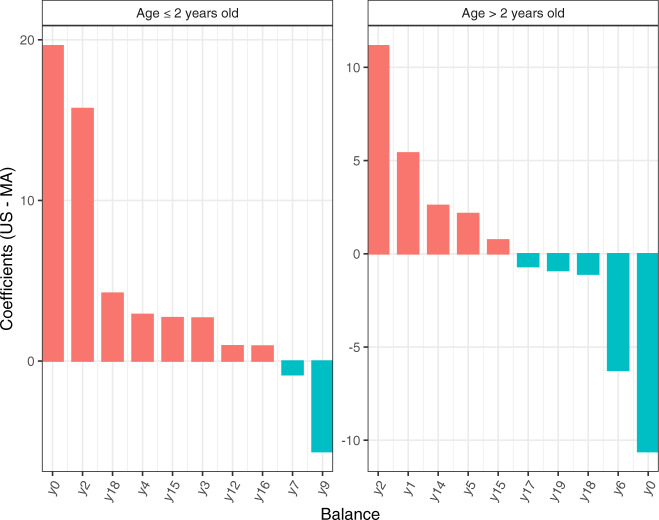
Waterfall plot visualizing coefficient (US: MA) for top 20 balances identified by gneiss using the global gut microbiota data^60^. We subset the dataset to subjects whose nationality is Malawi (MA) and USA (US), with sample size (MA:US) = (47:50) for the group of age ≤2 years old, and (MA:US) = (36:260) for the group of age >2 years old. The columns of the plot represent coefficients, and the rows of the plot represent balances. BH procedure^57^ was applied to correct for multiple comparisons, and coefficients with FDR corrected *p*-values < 0.05 are discarded.

We illustrate gneiss using the global gut data^60^ discussed earlier in this paper using Malawi (MA, *n*_1_ = 114) and the USA (US, *n*_2_ = 317) data. Gneiss identified different trends among various balances (Fig. 6). For example, balance *y*0 is detected to increase in US as compared to MA for subjects who are ≤2 years old; It is in a reverse direction for subjects who are >2 years old. One caveat to keep in mind is that the components of balances are not necessarily the same across different data sets. The first balance *y*0 for the younger generation (age ≤ 2 years old) consists of 642 taxa in the numerator (the left subtree) and 31 taxa in the denominator (the right subtree); On the other hand, *y*0 for the older group (age >2 years old) has 655 taxa in the numerator and 18 taxa in the denominator. It is important to note gneiss is not designed to infer changes in abundance for each individual taxon, however, it can answer questions such as whether the absolute abundances of taxa in the numerator of *y*0 on average have increased or decreased as compared to those in the denominator.

### LEfSe

Linear Discriminant Analysis Effect Size (LEfSe)^67^ is specifically designed for group comparisons of microbiome data with a particular focus on detecting change in relative abundance between two or more groups of samples with biological consistency. Important statistical and computational steps implemented in LEfSe are as follows:

1. For each taxon, test whether its observed abundances in different groups are differentially distributed using Kruskal–Wallis test.
2. (Optional, only if subgroups are defined) Discard taxa which are not statistically significant in step 1 (e.g. *p*-value > 0.05). The pairwise Wilcoxon test is then applied to retain taxa. A taxon is not retained for further consideration if it is not significant in every pairwise comparison (e.g. *p*-value > 0.05 for at least one pairwise comparison) or if the signs of test statistics are not equal among all comparisons.
3. After feature selection, a Linear Discriminant Analysis (LDA) model is built with the group label as the dependent variable and observed abundance of taxa selected in above step, subgroup label, and demographic features as independent variables. This model is used to calculate the effect size for each taxon. This effect size serves as the average of each taxon’s variability and discriminatory power.
4. Finally, the LDA score for each taxon is obtained by computing the logarithm (base 10) of the effect size after being scaled in the [1, 10^6^] interval. The rank for each taxon is assigned based on the corresponding LDA score and further feature selection could be achieved by setting a threshold (e.g. 2.0) for LDA scores.

By its construction, LEfSe method is more a discriminant analysis method rather than a DA method. Unlike the DA analysis methods discussed earlier in this paper, LEfSe is more focused on investigating the relationship among microbial profiles and an outcome or phenotype (Step 3). More precisely, LEfSe tries to quantify the magnitude of the effect size of such associations between microbial profiles (e.g. a set of taxa) and the outcome of interest.

## Discussion

Microbiome studies are becoming very popular in biomedical sciences. As new scientific questions emerge, so do new statistical and computational methods of analysis. This is a very rapidly growing area of research with new statistical methods being developed on a regular basis. Hence an up-to-date comprehensive review of the statistical methods in the field is a challenging problem. This is particularly true with methods for DA analysis. A number of methods exist in the literature and each method has its own strengths and weaknesses. One of the challenges in evaluating the performance of various methods is that not all methods are designed to test statistical hypotheses regarding the same parameter. Some methods are designed for testing hypotheses regarding the relative abundance, while others are designed for testing hypothesis regarding absolute abundance. If a simulation study is designed for testing hypothesis regarding absolute abundance then methods for relative abundance parameter may show an inflated FDR and vise versa. A related problem is that often researchers use the terms “relative abundance” and “absolute abundance in a unit volume” interchangeably. This makes the simulation studies difficult to interpret. Therefore journals and researchers should make the terminology precise. In this paper, simulation studies were set-up to compare FDR and power of various methods when testing hypotheses regarding absolute abundance of taxa in a unit volume of a tissue.

We performed simulation studies using the log-normal distribution for modeling abundances. Consistent with the findings of^20^, ANCOM and ANCOM-BC control the FDR at the desired nominal level for most configurations while competing well with all procedures in terms of the overall power. The only situations where ANCOM as well as ANCOM-BC fail to control FDR is when the sample sizes are very small, such as <10^20^. All other methods considered in this paper tend to inflate FDR for all sample sizes and their FDR gets worse with the sample size increases^20^. This is because, under the null hypothesis, each of these methods is biased away from zero. This bias increases with sample size. Hence the FDR increases with sample size.

While ANCOM and ANCOM-BC have very similar operating characteristics in terms of FDR and power, ANCOM-BC is computationally simpler and faster to implement because unlike ANCOM it requires only *m* linear regression fits rather than $\frac{m\;\times\,(m\,-\,1)}{2}$ models fits needed by ANCOM. Secondly, unlike ANCOM, ANCOM-BC provides individual p-values and confidence intervals of pairwise difference in mean abundance for each taxon. Among the methods available today, ANCOM-BC is the only procedure that provides valid p-values and confidence intervals. Furthermore, since ANCOM-BC is based on a regression model framework, it can easily be extended to repeated measures/longitudinal data covariate adjustments.

## Supplementary information

Supplementary Information

## Data Availability

DNA sequences from the global gut microbiota study^60^ can be found in MG-RAST https://www.mg-rast.org/index.html server under search string “mgp401” for Illumina V4-16S rRNA; feature table, metadata, and taxonomy of the diet swap data^68^ is available in the microbiome^69^ R package http://microbiome.github.com/microbiome.

## Code availability

All datasets and analysis scripts can be found under https://github.com/FrederickHuangLin/Microbiome-Review-Code-Archive.
