# Supplementary material for: Analysis of microbial compositions: a review of normalization and differential abundance analysis
Source: NPJ Biofilms Microbiomes. 2020 Dec 2;6:60. doi: 10.1038/s41522-020-00160-w (PMC7710733; doi:10.1038/s41522-020-00160-w)
Supplement: Supplementary file 1 — Supplementary Information [file 41522_2020_160_MOESM1_ESM.pdf]

Supplementary Information

Analysis of microbial compositions: A review of normalization and differential abundance analysis

Lin et al.

Supplementary Notes

Simulation settings

Fig. 3

(a) Nominal level = 0.05

(b) Number of simulations = 1

(c) Sample size: $n_1 = n_2 = n_3 = 30$

(d) Number of taxa: $m = 200$

(e) Proportion of differentially abundant taxa = 25%

(f) Proportion of structure zeros = 0% out of non-differentially abundant taxa

(g) Absolute abundance in the ecosystem: $\log A_{ij} = b_0 + b_i^T x_j + e_i$.

  (i) $b_0 = \log 50$ represents low abundant taxa, $b_0 = \log 200$ represents medium abundant taxa, and $b_0 = \log 10,000$ represents high abundant taxa. The proportions of low, median, and high abundant taxa are set to be $60\%, 30\%, 10\%$

  (ii) $x_j = (x_{j1}, \ldots, x_{jp})^T$ are covariates for the jth sample; $b_i = (b_{i1}, \ldots, b_{ip})^T$ are the corresponding coefficients for x_j. b_i are set to follow $\mathrm{U}(0.1, 1) \cup \mathrm{U}(1, 10)$ for differentially abundant taxa

  (iii) $e_i \sim N(0, \frac{1}{exp(b_0)})$

(h) Observed abundance from a sample:

  (i) Library sizes across samples: $O_{\cdot j} = p_j \max(A_{\cdot j})$, where $p_j \sim \frac{1}{\mathrm{U}(5,10)}$

  (ii) $O_{ij} \sim \mathrm{BIN}(O_{\cdot j}, \gamma_{ij} = \frac{A_{ij}}{A_{\cdot j}})$

Fig. 4

Simulation settings are the same as Fig. 3 except that:

(b) Number of simulations = 100

(c) Sample size: $n_1 = n_2 = 30$

(e) Proportion of differentially abundant taxa = $5\%, 15\%, 25\%$

(f) Proportion of structure zeros = 20% out of non-differentially abundant taxa

(h) Observed abundance from a sample:

  (i) Library sizes across samples: $O_{\cdot j} = p_j \max(A_{\cdot j})$, where $p_j \sim \frac{1}{\mathrm{U}(10,50) \cup \mathrm{U}(100,500)}$

  (ii) $O_{ij} \sim \mathrm{BIN}(O_{\cdot j}, \gamma_{ij} = \frac{A_{ij}}{A_{\cdot j}})$
